# The Impact of Intravascular Ultrasound Use on 1-Year Outcomes After Infrapopliteal Endovascular Intervention

**DOI:** 10.1016/j.jscai.2024.102509

**Published:** 2025-03-18

**Authors:** Daniel J. Snyder, Robert S. Zilinyi, Takehiko Kido, Danial Saleem, Isaac Dreyfus, Samuel Bruce, Elena Wadden, Zachary Girvin, Matthew T. Finn, Ajay J. Kirtane, Akiko Maehara, Sanjum S. Sethi, Sahil A. Parikh

**Affiliations:** a Division of Cardiology, Department of Medicine, Columbia University Irving Medical Center, New York, New York; b Division of Cardiology, Department of Medicine, University of California Los Angeles, Los Angeles, California; c Department of Medicine, NewYork-Presbyterian/Columbia University Irving Medical Center, New York, New York

**Keywords:** infrapopliteal, intravascular ultrasound, restenosis, revascularization

## Abstract

**Background:**

Data supporting the use of intravascular ultrasound (IVUS) in aortoiliac and femoropopliteal endovascular intervention are becoming increasingly robust, but data in the infrapopliteal circulation remain limited. The aim of this study was to evaluate the association between IVUS use and 1-year outcomes after infrapopliteal intervention.

**Methods:**

All infrapopliteal endovascular interventions that occurred between 2018-2021 at a single academic medical center were retrospectively reviewed. Cases were separated into 2 cohorts based on the use of IVUS. Clinical characteristics, procedural data, and imaging were collected up to 1-year postintervention. The primary outcome of interest was 1-year binary restenosis. Secondary outcomes included rates of 1-year target lesion revascularization, amputation, major adverse limb events, and all-cause mortality.

**Results:**

One hundred patients with 142 lesions were included. IVUS was used in 23% of cases. Baseline characteristics, lesion characteristics, and treatment modalities did not differ significantly between cohorts. At 1 year, cases with IVUS utilization had significantly lower rates of binary restenosis (22% vs 57%; *P* = .004) and substantially lower rates of target lesion revascularization (13% vs 33%; *P* = .06). Rates of amputation, major adverse limb events, and mortality did not differ significantly. In multivariate analysis, lesions treated with IVUS were significantly less likely to have binary restenosis at 1 year when compared with angiography alone (odds ratio, 0.18; 95% CI, 0.05-0.62; *P* = .007).

**Conclusions:**

Intravascular ultrasound use was associated with lower rates of 1-year restenosis when compared to angiography alone, even after correcting for differences in clinical and procedural characteristics. These findings support the need for a prospective randomized trial evaluating the impact of IVUS use on outcomes after infrapopliteal endovascular intervention.

## Introduction

Intravascular ultrasound (IVUS) functions through the use of an ultrasound transducer located at the tip of a catheter which, when delivered endovascularly, provides a 360-degree view of a vessel.^1^ Although angiography has traditionally been the primary imaging modality utilized in peripheral endovascular intervention (PVI), its characterization of a vessel is limited by the fact that it only provides a 2-dimensional representation of a 3-dimensional object. The use of IVUS enables operators to more accurately evaluate reference vessel size, lumen diameter, plaque morphology, and plaque geometry.^2^

Although originally developed for PVI,^3^ the uptake of IVUS in the peripheral circulation has lagged behind the coronary circulation. This is likely due to a lack of randomized controlled trial (RCT) data^4^; although RCTs exist which demonstrate the benefit of IVUS in reducing the risk of stent thrombosis and target lesion revascularization (TLR) in the coronary circulation,^5^ data on the peripheral circulation are mostly limited to observational studies, with the first RCT demonstrating the benefit of IVUS use in reducing restenosis rates having been published recently.^6^ Despite this, existing observational data support the notion that the benefits seen in the coronary circulation extend to the peripheral circulation as well.^7^ These data have been enough to support recent consensus statements which recommend the use of IVUS in PVI.^8^^,^^9^

Within PVI, the data evaluating the utility of IVUS in infrapopliteal intervention are particularly sparse. The 5 existing retrospective studies together suggest that IVUS provides larger vessel diameter estimates than angiography, enabling the use of larger diameter balloons and being associated with improvements in wound healing rates and skin perfusion pressures postintervention.^10^, ^11^, ^12^, ^13^, ^14^ To the authors’ knowledge, no studies exist in the infrapopliteal circulation that evaluate the association between IVUS use and rates of binary restenosis or TLR up to 1-year postintervention. The goal of this study was to address this gap by retrospectively evaluating the association between IVUS use and 1-year binary restenosis and revascularization rates after infrapopliteal endovascular intervention. The authors hypothesized that IVUS use would be associated with lower rates of binary restenosis and revascularization at 1 year when compared to angiography alone.

## Methods

### Cohort definition

A retrospective analysis of all infrapopliteal endovascular interventions occurring at a single academic medical center between January 2018 and December 2021 was conducted. The initial review revealed 103 patients with 145 distinct lesions. Cases with missing data (n = 3, 2.9%) were excluded from the analysis, yielding a final study population of 100 patients with 142 lesions.

### Ethical statement

The institutional review board at the authors’ institution approved this study (protocol AAAT2472) and waived the requirement for individual consent.

### Variables collected

Variables collected fell into 1 of 4 categories: demographics, clinical characteristics, lesion characteristics, or procedural characteristics. Demographic variables collected included age, sex, self-identified race, self-identified ethnicity, and insurance status (Medicare, Medicaid, commercial, uninsured/self-pay, or other). Clinical characteristics included body mass index, active smoking, and a history of hypertension, diabetes, coronary artery disease, congestive heart failure, cerebrovascular disease, end-stage renal disease, and chronic obstructive pulmonary disease. Baseline ankle-brachial index (ABI) and Rutherford classification were also collected along with the use of aspirin, P2Y-12 inhibitors, statins, cilostazol, anticoagulants, angiotensin-converting enzyme inhibitors, and angiotensin receptor blockers. Lesion characteristics included lesion location (anterior tibial, posterior tibial, tibioperoneal trunk, peroneal, or dorsal pedal), angiographic lesion length, Trans-Atlantic Inter-Society Consensus classification, and angiographic calcification score. Angiographic calcification was scored as either focal, mild, moderate, or severe with focal being calcification on 1 side of the artery less than half the length of the lesion, mild being calcification on 1 side of the artery greater than half the length of the lesion, moderate being calcification on both sides of the artery less than half the length of the lesion, and severe being calcification on both sides of the artery greater than half the length of the lesion. Procedural characteristics collected included primary treatment type (percutaneous old balloon angioplasty, drug-coated balloon, cutting balloon, lithotripsy balloon, bare-metal stent, or drug-eluting stent), primary treatment diameter, primary treatment length, and use of atherectomy. The primary treatment type was determined via manual chart review of the procedure note.

### Intravascular imaging review

Intravascular ultrasound images were independently reviewed to extract information regarding reference vessel size, lumen diameter, plaque morphology, and plaque geometry. Reference vessel diameter (RVD) was calculated as an average of the proximal and distal RVD, using minimum and maximum points at each end of the target vessel. Measurements of the proximal lumen area, proximal external elastic membrane (EEM) area, proximal internal elastic membrane (IEM) area, distal lumen area, distal EEM area, and distal IEM area were also extracted along with the proximal and distal plaque burden (%). In the diseased segment, minimum lumen area, plaque burden (%), EEM area, and IEM area were collected. Plaque composition was characterized as calcific, lipidic, or fibrous, and plaque geometry was classified as eccentric or concentric. When calcium was present, the morphology was characterized (eg, deep vs superficial, nodular) and the maximum calcium angle was calculated. IVUS images were also reviewed to extract information regarding procedural results and the presence of postprocedural complications.

### Outcome measures

The primary outcome of interest was binary restenosis. This was assessed at 6 months and 1-year postintervention and was defined as ≥50% stenosis on duplex ultrasound (eg, a peak systolic velocity ratio of ≥2.4) or angiography.^15^ All available duplex ultrasound and angiographic images were independently reviewed up to 1-year postintervention to identify lesions that met binary restenosis criteria. Secondary outcomes of interest assessed immediately postintervention included the following: (1) technical success rate, defined as residual stenosis ≤30% postintervention, (2) postprocedural complication rate, (3) mean improvement in ABI, (4) and median improvement in Rutherford classification. Postprocedural complications included access site complications (hematoma, occlusion, pseudoaneurysm, or fistula), thrombosis, embolization, target lesion dissection, and local perforation. Secondary outcomes of interest assessed at the 6-month and 1-year interval included the following: (1) TLR, (2) amputation, (3) major adverse limb event (MALE), defined as the need for TLR or major amputation above the level of the ankle, and (4) all-cause mortality.

### Statistical analysis

All statistical analyses were performed using SAS version 9.4 (SAS Institute). Two cohorts were created based on whether IVUS was utilized as an adjunctive imaging modality during the case or not. Demographics, clinical characteristics, lesion characteristics, and procedural characteristics were compared between cohorts using univariate analysis. Categorical variables were expressed as frequency (percentage) and compared using χ^2^ tests or Fisher exact tests when table frequencies were less than 5. Continuous variables were expressed as mean ± SD or median (IQR) and compared using Student’s *t* tests or Mann-Whitney *U* tests depending on the normality of data distribution. Descriptive analyses were performed using the IVUS data. Next, the primary and secondary outcomes of interest were compared between cohorts using univariate analysis. Survival analysis using Kaplan-Meier curves and log-rank testing was utilized to compare binary restenosis and TLR rates between cohorts up to 1-year postintervention. Finally, binary logistic regression was utilized to determine if IVUS use was a predictor of binary restenosis and TLR at 1-year. Variables were included in the multivariate models if they were found to be significant in univariate analysis (ie, *P* < .05). Results of logistic regression modeling were presented as odds ratios and 95% CI. Given survival time was available, Cox proportional hazard models were also created to determine whether IVUS use was a significant predictor of binary restenosis and TLR at 1 year. Subanalyses of patients who underwent atherectomy were also performed.

## Results

### Demographic data and clinical characteristics

A total of 100 patients with 142 distinct lesions were included in the study population; IVUS was used as an adjunctive imaging modality for 32 lesions (23%) (Table 1). When comparing baseline characteristics between cohorts, there were no significant differences with respect to demographics, clinical characteristics, preoperative medications, preintervention ABI, or preintervention Rutherford classification. Rutherford 3 patients were treated in the setting of multilevel disease.

**Table 1:** Baseline characteristics of the study population.

Variable	IVUS + angiography-guided (n = 32)	Angiography-guided (n = 110)	*P* value
Age, y	72.1 ± 13.2	69.9 ± 11.6	.35
Male sex	22 (68.8%)	80 (72.7%)	.66
Race			.16
White	15 (46.9%)	37 (33.6%)	
Black or African American	3 (9.4%)	25 (22.7%)	
Other or unknown^a^	14 (43.8%)	48 (43.6%)	
Hispanic or Latino	16 (50.0%)	48 (43.6%)	.52
Body mass index, kg/m^2^	24.5 ± 6.60	26.6 ± 4.95	.10
Current smoker	5 (15.6%)	18 (16.4%)	.92
Comorbidities			
Hypertension	27 (84.4%)	101 (91.8%)	.21
Diabetes	26 (81.3%)	88 (80.0%)	.88
Coronary artery disease	19 (59.4%)	73 (66.4%)	.46
Congestive heart failure	6 (18.8%)	25 (22.7%)	.63
Cerebrovascular disease	5 (15.6%)	20 (18.2%)	.74
End-stage renal disease	4 (12.5%)	14 (12.7%)	1
Chronic obstructive pulmonary disease	8 (25.0%)	14 (12.7%)	.1
Preoperative medications (%)			
Aspirin	22 (68.8%)	86 (78.2%)	.27
P2Y12 inhibitor	17 (53.1%)	63 (57.2%)	.68
Statin	30 (93.8%)	99 (90.0%)	.52
Anticoagulant	11 (34.4%)	27 (24.6%)	.27
ACE-inhibitor or ARB	14 (43.8%)	61 (55.5%)	.24
Cilostazol	2 (6.3%)	9 (8.2%)	1
Ankle-brachial index	0.70 ± 0.39	0.84 ± 0.41	.24
Rutherford classification			.31
3	10 (31%)	18 (16%)	
4	4 (13%)	20 (18%)	
5	14 (44%)	55 (50%)	
6	4 (13%)	17 (16%)	

Values are mean ± SD or n (%).

ACE, angiotensin-converting enzyme; ARB, angiotensin receptor blocker; COPD, chronic obstructive pulmonary disease; IVUS, intravascular ultrasound.

a This category included patients identifying as Asian (n = 0), Native Hawaiian/Pacific Islander (n = 2), 2 or more races (n = 15), or unknown/other races (n = 45).

### Angiographic lesion characteristics

The distribution of lesions within the infrapopliteal circulation (eg, anterior tibial, posterior tibial, etc) did not differ significantly between cohorts (Table 2). The Trans-Atlantic Inter-Society Consensus classification and lesion length were both similar (both *P* > .1). In the angiographic assessment, lesions treated with IVUS had substantially less calcification when compared to lesions treated with angiography alone (severe calcification, 68% vs 88%; *P* = .05).

**Table 2:** Lesion characteristics.

Variable	IVUS + angiography-guided (n = 32)	Angiography-guided (n = 110)	*P* value
Lesion location			.37
Anterior tibial	11 (34.4%)	39 (35.5%)	
Tibioperoneal trunk	11 (34.4%)	23 (20.9%)	
Posterior tibial	4 (12.5%)	26 (23.6%)	
Peroneal	6 (18.8%)	19 (17.3%)	
Dorsal pedal	0 (0.0%)	3 (2.7%)	
Angiographic lesion length, mm	115 ± 71.4	115 ± 82.8	.99
TASC classification^a^			.12
A	3 (12.5%)	2 (2.4%)	
B	0 (0.0%)	0 (0.0%)	
C	7 (29.1%)	23 (27.4%)	
D	14 (58.3%)	59 (70.2%)	
Calcification score^b^			.05
Focal	1 (4.6%)	1 (1.9%)	
Mild	0 (0.0%)	2 (3.9%)	
Moderate	6 (27%)	3 (5.8%)	
Severe	15 (68%)	46 (88%)	

Values are mean ± SD or n (%).

IVUS, intravascular ultrasound; TASC, Trans-Atlantic Inter-Society Consensus.

a Thirty-four cases had missing TASC classification data; the frequency of missing cases did not differ significantly between groups (8 [25%] IVUS-guided cases vs 26 [24%] angio-guided cases, *P* = .87).

b Sixty-eight cases had missing calcification scores; the frequency of missing cases did not differ significantly between groups (10 [31%] IVUS-guided cases vs 58 [53%] angio-guided cases, *P* = .14).

### IVUS findings

IVUS images were available for 26 of the 32 cases in the IVUS cohort (81%). The findings are summarized in Table 3. The average IVUS-derived RVD was 3.6 ± 0.7 mm (anterior tibial, 3.3 ± 0.5 mm; tibioperoneal trunk, 4.2 ± 0.7 mm; posterior tibial, 3.2 ± 0.5 mm; peroneal, 3.1 ± 0.3 mm). Average proximal and distal lumen areas were calculated at 5.7 ± 2.3 mm^2^ and 5.5 ± 2.4 mm^2^, respectively, with plaque burdens of 34% ± 15% and 30% ± 14%, respectively. In the diseased vessel segment, the mean lesion minimum lumen area was 3.3 ± 1.9 mm^2^, with an average plaque burden of 54% ± 11%. The distribution of eccentric and concentric plaque was fairly equal (46% vs 42%, respectively). Calcium was identified in 18 of the 26 lesions (69%), with superficial calcification present in 6 (23%) and deep calcification present in 16 (62%). The average maximum calcification angle was 158°, with 6 lesions (23%) having a calcium angle ≥180°. Calcified nodules were identified in 2 lesions (8%).

**Table 3:** Intravascular ultrasound findings.

Variable	n = 26
Reference vessel diameter, mm	3.6 ± 0.7
Anterior tibial	3.3 ± 0.5
Tibioperoneal trunk	4.2 ± 0.7
Posterior tibial	3.2 ± 0.5
Peroneal	3.1 ± 0.3
Proximal lumen area, mm^2^	5.7 ± 2.3
Proximal EEM area, mm^2^	11.7 ± 5.2
Proximal IEM area, mm^2^	10.0 ± 4.6
Proximal plaque burden, %	34 ± 15
Distal lumen area, mm^2^	5.5 ± 2.4
Distal EEM area, mm^2^	10.0 ± 3.9
Distal IEM area, mm^2^	8.5 ± 3.4
Distal plaque burden, %	30 ± 14
Lesion minimum lumen area, mm^2^	3.3 ± 1.9
Lesion EEM area, mm^2^	10.0 ± 4.8
Lesion IEM area, mm^2^	8.8 ± 4.3
Lesion plaque burden, %	54 ± 11
Eccentric plaque	12 (46%)
Concentric plaque	11 (42%)
Fibrous plaque	21 (81%)
Lipidic plaque	3 (12%)
Calcification	18 (69%)
Superficial calcium	6 (23%)
Deep calcium	16 (62%)
Calcified nodule	2 (8%)
Maximum calcium angle, °	158 ± 116
Calcium angle ≥180°	6 (23%)

Values are mean ± SD or n (%).

EEM, external elastic membrane, IEM, internal elastic membrane.

### Treatment strategies

The primary treatment strategy (eg, drug-coated balloon, drug-eluting stent, etc) did not differ significantly between cohorts (Table 4). There were no significant differences in balloon or stent sizing between cases with IVUS use and cases treated using angiography alone. Lesions treated with IVUS guidance had a significantly higher frequency of atherectomy use than lesions treated with angiography guidance alone (21.9% vs 8.2%; *P* = .03).

**Table 4:** Univariate comparison of treatment strategies between IVUS-guided and angiography-guided cases.

Variable	IVUS + angiography-guided (n = 32)	Angiography-guided (n = 110)	*P* value
Treatment type^a^^,^^b^			.08
POBA	17 (53.1%)	78 (74.3%)	
DCB	5 (15.6%)	7 (6.7%)	
Cutting balloon	1 (3.1%)	3 (2.9%)	
Lithotripsy balloon	7 (21.9%)	8 (7.6%)	
Bare-metal stent	0 (0.0%)	3 (2.9%)	
Drug-eluting stent	2 (6.3%)	6 (5.7%)	
Unable to treat	0 (0.0%)	5 (4.6%)	.59
POBA diameter, mm	2.91 ± 0.57	2.84 ± 0.72	.68
DCB diameter, mm	4.40 ± 0.89	4.36 ± 1.20	.94
Stent diameter, mm	3.25 ± 0.35	3.41 ± 1.07	.85
POBA treatment length, mm	137 ± 64.9	120 ± 74.6	.41
DCB treatment length, mm	108 ± 45.5	87.1 ± 34.9	.39
Stent treatment length, mm	43 ± 7.1	50.3 ± 29.3	.75
Atherectomy performed	7 (21.9%)	9 (8.2%)	.03

Values are mean ± SD or n (%).

DCB, drug-coated balloon; IVUS, intravascular ultrasound; POBA, plain old balloon angioplasty.

a Cases where the intervention was aborted were excluded from this analysis.

b All cases where stents were used (n = 11) used single stents; predilation and postdilation balloon diameters were excluded given the infrequency of stent use (7.7%).

### Primary and secondary outcomes

In univariate analysis, lesions treated with IVUS guidance had similar technical success rates and postprocedural complication rates compared to lesions treated with angiography guidance alone (Table 5). Improvements in ABI and Rutherford classification were also similar between cohorts. At 6 months, rates of binary restenosis and TLR were lower in the IVUS cohort (16% vs 34.6% and 12% vs 19.8%, respectively) but this difference was not statistically significant (both *P* > .08). Rates of amputation, MALE, and all-cause mortality were similar at 6 months. By 1 year, rates of binary restenosis were significantly lower for lesions treated with IVUS guidance when compared to lesions treated with angiography alone (21.7% vs 56.7%, *P* = .004). The difference in TLR rates between cohorts approached but did not reach statistical significance at 1 year in univariate analysis (13.1% vs 33.3%, *P* = .06) or multivariable analysis (see Table 6). Notably, when analyzing rates of binary restenosis and TLR among patients who received atherectomy, IVUS use was still associated with a significantly lower frequency of binary restenosis and a substantially lower frequency of TLR (*P* = .01 and *P* = .05, respectively). Rates of amputation, MALE, and all-cause mortality did not differ significantly at 1 year. Differences in freedom from binary restenosis rates and TLR rates over time are depicted in the Kaplan-Meier curves in the Central Illustration. In multivariate analysis adjusting for differences in baseline characteristics, cases treated with IVUS guidance had significantly lower odds of binary restenosis at 1 year than cases treated with angiography guidance alone (odds ratio, 0.21; 95% CI, 0.06-0.76; *P* = .02) (Table 6). A similar effect was also demonstrated using Cox proportional hazards modeling, with IVUS use carrying a hazard ratio of 0.27 for binary restenosis (95% CI, 0.11-0.71; *P* = .01) after correcting for differences in baseline characteristics. Cox proportional hazards modeling did not demonstrate a significant difference in TLR rates with IVUS use.

**Table 5:** Univariate comparison of outcomes between IVUS-guided and angiography-guided cases.

Variable	IVUS + angiography-guided (n = 32)	Angiography-guided (n = 110)	*P* value
Technical success rate	32 (100%)	102 (92.7%)	.11
Complication rate	1 (3.1%)	14 (12.7%)	.19
ABI improvement	0.25 ± 0.20	0.14 ± 0.31	.37
Median Rutherford improvement	1 (0-5)	0 (0-3)	.36
6-month outcomes^a^			
Restenosis	4 (16%)	28 (34.6%)	.08
Reintervention	3 (12%)	16 (19.8%)	.55
Amputation	2 (8.0%)	8 (9.9%)	1
MALE	5 (20.8%)	18 (22.2%)	.81
All-cause mortality	0 (0.0%)	5 (6.2%)	.59
1-year outcomes^b^			
Restenosis	5 (21.7%)	34 (56.7%)	.004
Reintervention	3 (13.1%)	20 (33.3%)	.06
Amputation	4 (17.4%)	10 (16.7%)	1
MALE	5 (21.7%)	24 (40%)	.12
All-cause mortality	1 (4.4%)	10 (16.7%)	.28

Values are mean ± SD, median (IQR), or n (%).

ABI, ankle-brachial index; IVUS, intravascular ultrasound; MALE, major adverse limb events (revascularization or major amputation).

a 35 patients were lost to follow-up by 6 months; the frequency of patients lost to follow-up did not significantly differ between cohorts (7 [21.9%] vs 28 [25.5%], *P* = .68).

b 59 patients were lost to follow-up by 1 year; the frequency of patients lost to follow-up did not significantly differ between cohorts (9 [28.1%] vs 50 [45.4%], *P* = .08).

**Table 6:** Logistic regression and Cox regression analyses evaluating the impact of IVUS use on binary restenosis and TLR rates at 1 year.

Variable	Unadjusted odds ratio/hazard ratio (95% CI)^a^	*P* value	Adjusted odds ratio/hazard ratio (95% CI)^b^	*P* value
Logistic regression				
1-year restenosis				
IVUS use	0.21 (0.07-0.65)	0.01	0.21 (0.06-0.76)	.02
1-year TLR				
IVUS use	0.28 (0.07-1.05)	0.06	0.26 (0.06-1.08)	.06
Cox regression				
1-year restenosis				
IVUS use	0.28 (0.10-0.71)	0.01	0.27 (0.11-0.71)	.01
1-year TLR				
IVUS use	0.32 (0.10-1.08)	0.07	0.33 (0.10-1.11)	.08

IVUS, intravascular ultrasound, TLR, target lesion revascularization.

a Odds ratios provided for logistic regression results and hazard ratios provided for Cox regression results.

b Models were created using variables that were found to be significant (*P* < .05) in univariate analysis.

**Central Illustration:**
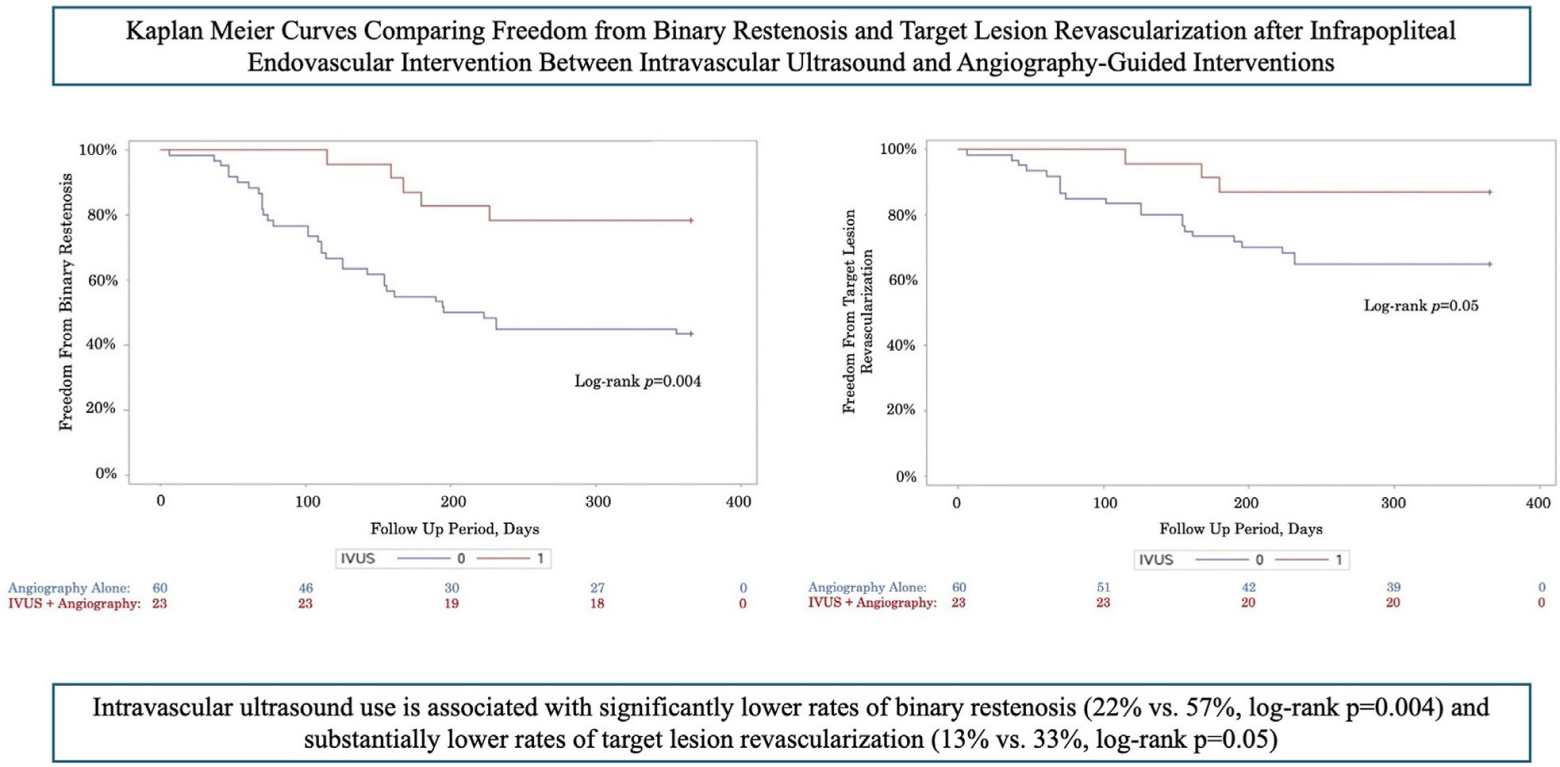
Depiction of Kaplan-Meier analyses showing significantly lower rates of binary restenosis and substantially lower rates of target lesion revascularization when intravascular ultrasound (IVUS) was utilized to guide infrapopliteal endovascular intervention as opposed to angiography alone.

## Discussion

Despite numerous advances in endovascular technologies for peripheral arterial disease (PAD) treatment over the past 30 years, binary restenosis rates remain high after infrapopliteal intervention.^16^, ^17^, ^18^, ^19^ Developing strategies to combat rates of restenosis is critical in minimizing the need for reintervention or amputation and maintaining the viability of endovascular intervention as a treatment option for patients with infrapopliteal chronic limb-threatening ischemia (CLTI).^20^ The results of this study demonstrate that in patients with infrapopliteal PAD, rates of binary restenosis are significantly lower at 1 year when IVUS is utilized as an adjunctive imaging modality during the initial intervention.

To the authors’ knowledge, this is the first study to demonstrate an association between IVUS use and binary restenosis rates up to 1 year after infrapopliteal endovascular intervention. This finding builds upon previous literature on infrapopliteal circulation supporting an association between IVUS use and improved outcomes postintervention. Fujihara et al^14^ demonstrated using a population of 216 patients with infrapopliteal CLTI that IVUS use was associated with improved skin perfusion pressures postintervention which translated into significantly faster wound healing and significantly higher rates of complete wound healing at 3, 6, and 12 months. This was followed by a single-center retrospective cohort study by Soga et al^13^ which evaluated 155 patients with infrapopliteal CLTI and found that IVUS use was associated with significantly faster rates of wound healing (84 ± 55 days vs 135 ± 118 days) and higher rates of limb salvage without reintervention at 12 months. The finding of improved binary restenosis rates also coincides with previously published retrospective data on aortoiliac circulation, RCT data on femoropopliteal circulation, and large meta-analyses of prospective data on coronary circulation.^6^^,^^21^, ^22^, ^23^, ^24^, ^25^

The reason behind improved rates of binary restenosis with IVUS use is likely multifactorial. Preintervention, IVUS use helps operators better delineate plaque composition to determine whether vessel preparation is needed prior to angioplasty or stenting.^8^ In this study, despite having lower levels of calcification based on angiographic assessment, rates of atherectomy usage were >2.5x higher when IVUS was utilized. Although the benefit of adjunctive atherectomy has not been consistently demonstrated in infrapopliteal PAD, several trials including CALCIUM 360, OPTIMIZE BTK, and LIPS2 suggest that there may be a benefit in rates of binary restenosis and TLR up to 1-year postintervention when orbital or laser atherectomy are used.^26^, ^27^, ^28^ Results of a subanalysis of the patients who received atherectomy in this study population suggest that lesion preparation does not solely explain the association of IVUS use and improved durability of the intervention, however, as the patients who had IVUS utilized in this subpopulation still had lower rates of binary restenosis and TLR than those who did not have IVUS utilized. Other lesion preparation techniques including intravascular lithotripsy were also higher in the IVUS cohort. Intraprocedurally, IVUS use helps optimize device sizing by providing more accurate RVDs.^8^ Although previous literature suggests that IVUS provides significantly larger RVD estimates than angiography which typically translates to larger balloon sizing,^10^, ^11^, ^12^, ^13^, ^14^ this study found no difference in balloon sizing between cohorts. Although it is possible that angiographic vessel diameters were smaller in the IVUS cohort and that IVUS still enabled upsizing, this study was not designed to evaluate this variable. The recently published IVUS-DCB trial also suggested that even when primary device sizing did not differ, IVUS enabled the use of larger preballoon and postballoon diameters for lesion preparation and device optimization, which this study was also not designed to evaluate.^29^ Finally, postintervention, IVUS use facilitates the identification of complications that can be addressed prior to the end of the case including dissection, stent underexpansion, and stent malapposition. This study was not powered to assess for these outcomes but did not demonstrate any difference in complication rates postintervention when IVUS was utilized.

Although rates of binary restenosis were significantly lower in the IVUS cohort, this did not result in a significant difference in rates of TLR. The same finding was noted by Allan et al^6^ in an RCT investigating the impact of IVUS on binary restenosis and revascularization rates 1 year after femoropopliteal endovascular intervention. As suggested in this study, it is possible that because the decision for revascularization is driven by clinical factors instead of imaging criteria, a longer follow-up interval is needed to include when cases of restenosis transition to the point of producing clinical symptoms. The Kaplan-Meier curves from this study data support this notion, as revascularization rates lagged behind restenosis rates but diverged in a very similar manner, almost reaching statistical significance at 1 year. Future research evaluating the impact of IVUS use on revascularization rates should consider extending the follow-up interval beyond 1 year to ensure appropriate evaluation of revascularization needs. This distinction is critically important not only in demonstrating the clinical utility of IVUS but also in evaluating its cost-effectiveness. Although IVUS represents a cost of ∼ $1100 to $1300 during the index procedure according to prior studies,^24^^,^^30^ a benefit in terms of revascularization would produce cost savings that far outweigh this upfront cost.

Despite encouraging evidence supporting the use of IVUS and recent consensus statements recommending the use of IVUS in PVI, rates of uptake across the country have been slow.^31^ At the authors’ primary institution, which houses interventionalists who specialize in the usage of IVUS, IVUS was only utilized in 23% of cases during the study period. This, although representing only a minority of cases, reflects a utilization rate almost 6x higher than the national inpatient usage rate of around 4%.^31^ There are several factors that are likely contributing to this slow rate of uptake, the most prominent of which is the lack of data in the space. Although this study provides some of the first observational data that suggest IVUS use improves binary restenosis rates, there are no RCTs substantiating that IVUS use impacts outcomes postintervention in the infrapopliteal circulation. Given that IVUS use requires an additional time investment and reimbursement is less favorable in the inpatient setting,^32^ interventionalists need to be provided with Level 1 evidence supporting the impact of IVUS on outcomes postintervention. Future research should substantiate the findings of this study in an RCT format.

### Limitations

The authors acknowledge several important limitations of this study. First, this study utilized data from a single academic medical center with an endovascular service that specializes in the use of IVUS. Recent analyses have shown that the frequency of IVUS use is extremely variable across the United States, with some centers having little experience with the modality and other, typically ambulatory centers using it in up to 25% of cases.^31^ This may hamper the generalizability of the results to centers with less experience with IVUS. The data utilized for this study were also collected retrospectively. This design does not allow for evaluation of whether IVUS use changed the operator’s decision regarding primary treatment type, device size, or need for plaque modification. Prior research suggests that this occurs in almost 80% of cases, which may be a factor driving the differences seen in outcomes.^6^ This study also demonstrated differences in angiographic calcification severity between cohorts but given the extent of missing data for this variable, the authors were unable to adjust for this variable in the multivariable analyses. Previous literature has suggested that severe calcification is associated with worse postprocedural outcomes, and this may have impacted the results of this study as well.^33^^,^^34^

## Conclusion

This study adds to a sparse set of literature on infrapopliteal circulation evaluating the association between IVUS use and postintervention outcomes. Results suggest that IVUS use is associated with lower rates of binary restenosis at 1 year when compared to the use of angiography alone, even after correcting for differences in clinical and procedural characteristics. These findings suggest the need for the development of an RCT evaluating the impact of IVUS use on procedural outcomes after infrapopliteal endovascular intervention.
